# Renal cell carcinoma metastasis to the ciliary body responds to proton beam radiotherapy: a case report

**DOI:** 10.1186/1752-1947-5-345

**Published:** 2011-08-03

**Authors:** Tarek Alasil, Bahram Khazai, Lilia Loredo, Michael E Rauser

**Affiliations:** 1 Department of Ophthalmology, Loma Linda University, Loma Linda, CA 92354, USA

## Abstract

**Introduction:**

We report an unexpected presentation of metastatic renal cell carcinoma (RCC) to the ciliary body and an interesting response to proton beam radiotherapy.

**Case presentation:**

We encountered a case of angle-closure glaucoma as the initial presentation of ocular metastasis to the ciliary body in a 65-year-old Caucasian man who had undergone right radical nephrectomy for RCC 15 years earlier. He underwent YAG (yttrium aluminium garnet) laser peripheral iridotomy while further metastatic workup took place. His condition was eventually diagnosed as stage IV metastatic RCC of the clear cell type and involved multiple sites, including the ciliary body, brain, lungs, liver, and pancreas. The progression of RCC metastasis to the ciliary body was studied for 16 months. The ciliary body mass continued to grow despite systemic treatment with temsirolimus and interleukin-2 and intravitreal injections of bevacizumab. The tumor size peaked at 6.11 × 6.06 mm before the start of proton therapy, which reduced the tumor size to 5.07 × 4.39 mm.

**Conclusions:**

RCC can produce metastases involving unusual sites many years after resection of the primary tumor. Proton therapy was found to be effective in treating RCC metastasis to the ciliary body in settings in which other treatment modalities failed.

## Introduction

Renal cell carcinoma (RCC) accounts for approximately 85% of primary renal neoplasms and represents approximately 3% of all adult malignancy [1]. The most common sites of metastasis are the lung (50%) and the bone (33%), but RCC has been documented to metastasize to every organ and site in the body by hematogenous spread. The interval between RCC systemic and ocular presentations varies and may become so prolonged as to obscure the relationship between the ocular metastasis and the primary RCC tumor [2]. In a pathologic survey, Ferry and Font [3] demonstrated that only seven out of 196 cases of ocular metastatic carcinoma originated from RCCs. Ocular metastases from RCC are most likely to involve the iris [4], ciliary body [5], and choroids, although eyelid and orbital metastases have been described [6].

We report a rare case of angle-closure glaucoma secondary to ciliary body metastasis in a patient who underwent radical nephrectomy for RCC 15 years earlier. Also, we review the different treatment modalities we used to treat the ocular metastasis.

## Case presentation

A 65-year-old Caucasian man presented to our emergency department and complained of sudden-onset blurry vision and pain in his right eye. He reported associated nausea and a right-sided headache. His medical history was significant for RCC, for which he had undergone right radical nephrectomy 15 years earlier. After the initial nephrectomy, he had completed 10 years of surveillance, after which he was thought to be disease-free.

**Figure 1:**
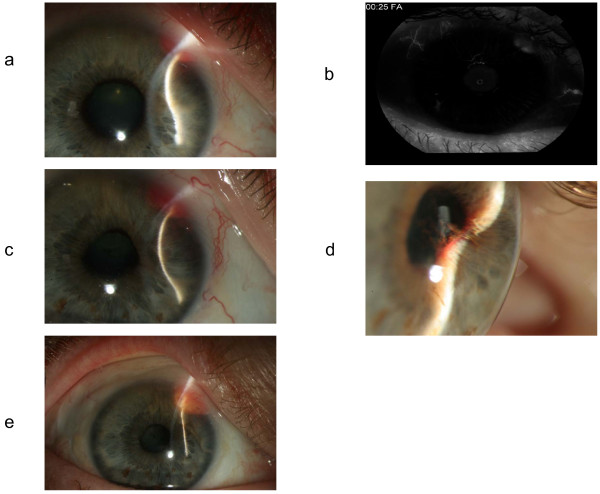
**(a) Slit-lamp exam (SLE) photograph of the right eye at presentation shows a red mass in the superior nasal aspect of the iris at one to three o'clock**. (b) Iris angiography of the right eye shows an area of hyperfluorescence over the area of the tumor. (c) SLE photograph of the right eye 10 months later (status after systemic treatment with temsirolimus and interleukin-2 and intravitreal injections of bevacizumab) shows that the red mass had grown. (d) SLE photograph delineates the mass-lens adhesions prior to proton beam radiotherapy. (e) SLE photograph of the right eye after proton radiotherapy shows a significant decrease in the size of the mass.

**Figure 2:**
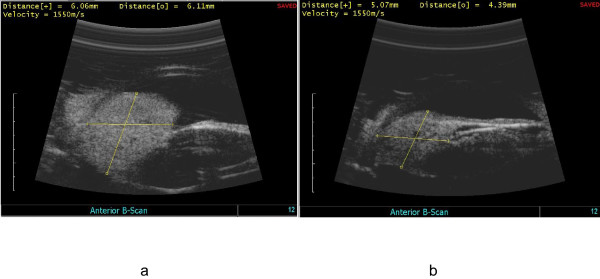
**(a) High-frequency immersion B-scan ultrasonography of the right eye reveals the ciliary body mass at a peak size of 6.11 × 6.06 mm**. (b) B-scan ultrasonography after proton radiotherapy reveals the regression of the ciliary body mass size to 5.07 × 4.39 mm.

At presentation, his best corrected visual acuities were 20/50 in his right eye (*oculus dexter*, or OD) and 20/20 in his left eye (*oculus sinister*, or OS). The intraocular pressures by applanation tonometry were 50 mm Hg OD and 14 mm Hg OS. The results of a left eye exam were unremarkable. A slit-lamp exam of his right eye demonstrated a red mass that was located in the superior nasal aspect of his iris at one to three o'clock and that was associated with dilated episcleral feeder vessels (Figure 1a). A gonioscopy showed a closed angle with the iris bowing forward to the superior nasal sector of the anterior chamber angle. A high-frequency immersion B-scan ultrasonography of his right eye revealed a ciliary body mass, which was pushing his iris forward toward his cornea (Figure 2a). An iris angiography of his right eye showed an area of hyperfluorescence over the area of his tumor (Figure 1b). A fundus examination of his right eye showed no evidence of choroidal or retinal involvement.

His condition was diagnosed as acute angle-closure glaucoma (AACG) secondary to a ciliary body mass in his right eye. He was started on timolol, acetazolmide, and latanoprost followed by a YAG (yttrium aluminium garnet) laser peripheral iridotomy to lower the intraocular pressure.

**Figure 3:**
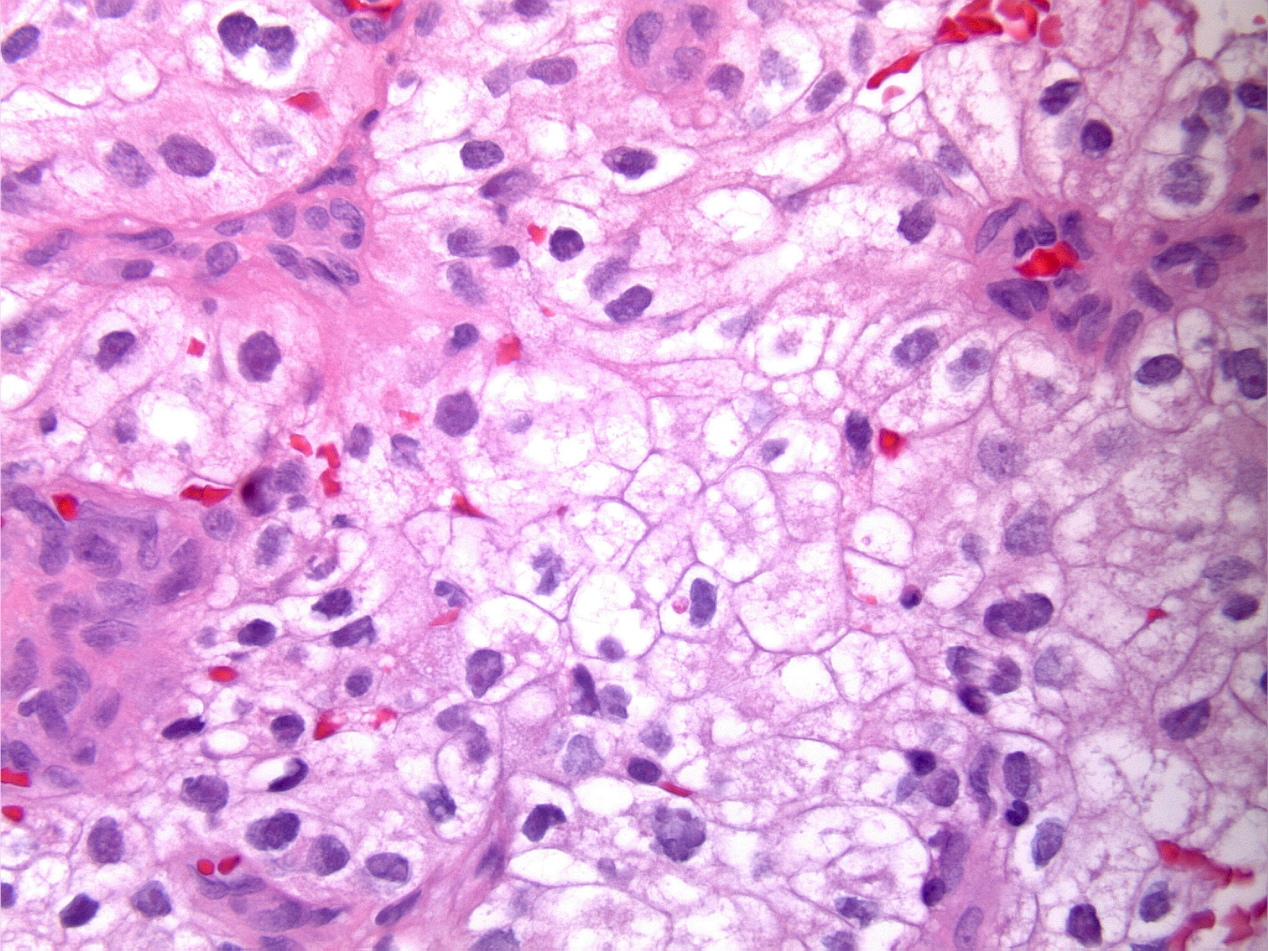
**Histopathologic image of optically clear cytoplasm, sharply outlined cell membranes, round to irregular nuclei, and prominent nucleoli**. Pathology findings are consistent with clear cell carcinoma, Fuhrman grade III (renal cell carcinoma metastasis).

The results of a further metastatic workup were consistent with a stage IV RCC that was of the clear cell type (Figure 3) and that involved the ciliary body, brain, lungs, liver, and pancreas.

The ciliary body mass continued to grow despite systemic treatment with temsirolimus (mammalian target of rapamycin inhibitor) [7] and interleukin-2 (IL-2) (Figure 1c), but the extraocular metastasis started to shrink. Later, two intravitreal injections of bevacizumab failed to slow the growth of the mass, and the tumor size peaked at 6.11 × 6.06 mm (Figure 2a). Some adhesions have developed between the mass and the lens (Figure 1d).

Lastly, proton radiotherapy was administered. Tantalum clips were placed to outline the posterior margin of the tumor. Our patient received a total of 30 cobalt gray equivalents (CGE) in three fractions given over the course of six calendar days. As a result, the tumor size decreased to 5.07 × 4.39 mm (Figures 1e and 2b). Later, our patient underwent an uneventful cataract extraction and an intraocular lens placement in his right eye. His current visual acuity in his right eye is 20/40.

## Discussion

The mechanism of ocular spread in RCC is presumed to take place through venous diffusion within the small chroidal vessels, and neoplastic cells travel as emboli [8]. Simultaneous bilateral iris metastases from RCC were described by Wizinski and colleagues [4]. Two cases of spontaneous disappearance of choroidal metastasis from RCC after nephrectomy have been described [9,10]. Shome and colleagues [11] reported a case of iris metastasis from RCC 14 months after a right nephrectomy. Mancini and colleagues [8] reported a case of left ciliary body metastasis in a 42-year-old man who had undergone a left radical nephrectomy for conventional RCC six years earlier. Our patient is unusual because, before he presented with AACG, he was thought to be disease-free for 15 years after nephrectomy but he was eventually discovered to have widespread RCC metastasis. Although the extraocular metastasis showed a reasonable response to systemic temsirolimus and IL-2, the ciliary body metastasis failed to do so. One theory may question the bioavailability of these systemic agents in the ciliary body circulation system.

Primary RCC produces angiogenic growth factors (such as basic fibroblastic growth factor and vascular endothelial growth factor), which are responsible for tumor growth, proliferation, metastases, and survival through high serum levels [12]. Unfortunately, our patient's tumor continued to grow despite intravitreal injections of bevacizumab (anti-vascular endothelial growth factor).

The prognosis of metastatic RCC is generally poor; median survival is 10 months and five-year survival is less than 5% [13,14]. Our patient has survived for 16 months with an extraocular response to the systemic temsirolimus and IL-2. His affected eye maintained visual acuity and the ciliary body mass regressed in response to the proton radiotherapy followed by successful cataract surgery.

Several urologists advocated RCC surveillance for at least 10 years after the initial nephrectomy. Others concluded that follow-up for life is reasonable [15-20]. Our case report would emphasize such extended surveillance as long as it is individualized and cost-conscious.

## Conclusions

We present an interventional case report in which the progression of RCC metastasis to the ciliary body was studied for 16 months. The response to different treatment modalities was investigated. The ciliary body mass continued to grow despite systemic treatment with temsirolimus and IL-2 and intravitreal injections of bevacizumab. The tumor size peaked at 6.11 × 6.06 mm before the start of proton therapy, which reduced the tumor size to 5.07 × 4.39 mm. To the best of our knowledge, this is the first case report of a ciliary body metastasis from RCC that responded to proton radiotherapy after other treatment modalities had failed.

## Consent

Written informed consent was obtained from the patient for publication of this case report and any accompanying images. A copy of the written consent is available for review by the Editor-in-Chief of this journal.

## Abbreviations

AACG: acute angle-closure glaucoma; IL-2: interleukin-2; OD: *oculus dexter *(right eye); OS: *oculus sinister *(left eye); RCC: renal cell carcinoma.

## Competing interests

The authors declare that they have no competing interests.

## Authors' contributions

TA analyzed and interpreted the patient data and wrote the manuscript. BK helped to gather the data and write the manuscript. LL was the radiation oncologist who treated our patient with proton beam radiotherapy. MER was the attending ophthalmologist who evaluated our patient, performed the bevacizumab intravitreal injections, and coordinated the proton beam radiotherapy. All authors have read and approved the final manuscript.
